# Relationships among self-esteem, depression and self-injury in adolescents: a longitudinal study

**DOI:** 10.3389/fpubh.2024.1406283

**Published:** 2024-05-15

**Authors:** Hui Lei, Jingru Xiong, Yuling Rao, Ting Zhu, Xiaocui Zhang

**Affiliations:** ^1^College of Education, Hunan Agricultural University, Changsha, China; ^2^Medical Psychological Center, The Second Xiangya Hospital, Central South University, Changsha, China; ^3^Medical Psychological Institute of Central South University, Changsha, China; ^4^National Clinical Research Center for Mental Disorders, Changsha, China

**Keywords:** NSSI, self-esteem, depression, adolescent, longitudinal study, cross-lagged analysis

## Abstract

**Objective:**

Non-suicidal self-injury is a widespread mental health concern among adolescents. This study aimed to examine the relationship between self-esteem, depression, and self-injury among adolescents using a longitudinal research design.

**Methods:**

The Self-Esteem Scale (SES), Child Depression Inventory (CDI), and Adolescent Self-Injury Scale (ASIS) were used to follow up 1,265 junior middle school students on three occasions with six-month intervals.

**Results:**

At all three time points, there were significant gender differences in self-esteem, depression, and self-injury. Self-esteem was negatively correlated with depression and self-injury at all three time points, while depression and self-injury were significantly positively correlated. Cross-lagged analysis revealed that self-esteem at Time 1 (T1) did not significantly predict self-injury at Time 2 (T2), but self-esteem (T2) significantly predicted self-injury at Time 3 (T3; *β* = −0.079, *p* < 0.05). Similarly, self-injury (T1) significantly predicted self-esteem (T2; *β* = −0.140, *p* < 0.001), and self-injury (T2) significantly predicted self-esteem (T3; *β* = −0.071, *p* < 0.01). Horizontal and longitudinal mediating analysis showed that depression served as a complete mediator in both the pathway from self-esteem to self-injury and from self-injury to self-esteem. Cross-lagged analysis showed that self-esteem (T1) significantly predicts depression (T2; *β* = −0.070, *p* < 0.05), which in turn predict self-injury (T3; *β* = 0.126, *p* < 0.001). Similarly, self-injury (T1) predicted depression (T2; *β* = 0.055, *p* < 0.05), which further predicted self-esteem (T3; *β* = −0.218, *p* < 0.001).

**Conclusion:**

The self-esteem, depression, and self-injury of adolescents are closely related; self-esteem and self-injury predict each other; self-esteem indirectly affects self-injury through depression; and self-injury indirectly affects self-esteem through depression. Based on the relationship of bi-directional prediction of self-esteem and self-injury mediated by depression, this study proposes a theoretical model of depression-mediated self-esteem and self-injury cycle.

## 1 Introduction

Non-suicidal self-injury (NSSI), abbreviated as self-injury, refers to the intentional and repeated act of causing harm to one’s own body without any suicidal intent. Common methods of harm include cutting, burning, biting hands, and pulling hair (1). NSSI is a widespread mental health concern among adolescents. In a large-sample study, 17.59% of adolescents were found to have committed at least one self-injury in the past 12 months (2). A meta-analysis of the detection rate of mental health problems in Chinese junior high school students between 2010 and 2020 found that the self-injury detection rate was 22% (3).

Previous studies have shown a strong relationship between self-esteem and self-injury. For example, a systematic review study showed a significant negative relationship between self-esteem and self-injury, that individuals with a history of self-injury had lower levels of self-esteem than individuals without a history of self-injury, and that low self-esteem was common characteristic among those who engage in self-injury or have a history of self-injurious behavior (4). Self-esteem can predict self-injury behavior 1 year later, with individuals having lower levels of self-esteem being more likely to engage in self-injury behavior after 1 year (5). A longitudinal study with 830 New Zealand adolescents examined the relationship using cross-lagged analysis and showed that self-esteem significantly negatively predicted self-injury, whereas self-injury was not a significant predictor of self-esteem (6).

There is a high correlation and comorbidity between depression and self-injury. The number of individuals engaging in self-injury among those with depressive disorders is significantly higher than among non-depressed individuals (7). Students with self-injurious behavior had significantly higher levels of depression than those without self-injurious behavior (8). A two-year follow-up study found that depression in the first year predicted the onset of self-injury but was not a significant predictor of the onset of self-injury in the second year (9). A systematic review also noted that depression in adolescents predicted the development of future self-injurious behavior (10). However, there is no unified conclusion on the predictive effect of self-injury on depression. Some studies have found that self-injury effectively predict subsequent depression (11, 12), but other studies have discovered that it cannot (13, 14).

The experiential avoidance model of self-injury suggests that individuals develop a specific emotional response when stimulated. Individuals tend to engage in avoidance through self-injury, which not only briefly alleviates their emotional response, but the emotional relief further reinforces their self-injurious behaviors, thus contributing to the persistence of self-injury. Self-injury is a non-adaptive behavior adopted by individuals to alleviate negative emotions (depression), However, whether the depressive emotions of individuals are truly alleviated after self-injury has not yet reached a consensus among researchers.

Regarding the relationship between self-esteem and depression, there are three perspectives: One perspective is based on the cognitive vulnerability model of depressive, which posits that self-esteem is a vulnerability factor for depression. The lower the level of self-esteem, the higher the level of depression. Low self-esteem can significantly predict depression (15, 16). The second perspective is based on the scar model, which suggests that low self-esteem is more often a result of depression rather than the cause of depression (17). The third view suggests that self-esteem and depression are causal and that there is a bidirectional predictive relationship between the two (18).

Previous research has shown that self-esteem, depression, and self-injury are closely related, but questions about how both self-esteem and depression affect self-injury, and how self-injury affects self-esteem and depression, remain unclear. Therefore, the present study utilized a longitudinal research design to explore the relationship between self-esteem, depression, and self-injury. First, although previous studies have confirmed that self-esteem is negatively related to self-injury, the effect of self-injury on self-esteem needs to be further confirmed. Based on previous research, the present study proposes Hypothesis 1: There is a bidirectional predictive effect of self-esteem and self-injury.

The cognitive vulnerability model of depression suggests that self-esteem may be a vulnerability factor for depression, where individuals with low levels of self-esteem are prone to developing depressive emotions. Once depressive emotions arise, individuals may adopt various coping mechanisms. The experiential avoidance model posits that when individuals experience negative emotions (depression), they may engage in self-injury as a rapid means of relief. Therefore, this study proposes Hypothesis 2: While self-esteem directly influences self-injury, it may also indirectly affect self-injury through depression, with depression serving as a mediator between self-esteem and self-injury.

In previous research, the focus has predominantly been on investigating the factors influencing self-injury, with few studies exploring the impact of self-injury on individuals’ emotions and cognition. The effect of self-injury on subsequent depression has not yet reached a consensus. The scar model suggests that the onset of depression can leave a lasting scar, causing negative impacts on the individual, specifically leading to a reduction in self-cognition. Therefore, this study proposes Hypothesis 3: While self-injury directly affects self-esteem, it may also indirectly influence self-esteem through depression, with depression serving as a mediator between self-injury and self-esteem.

**Figure 1:**
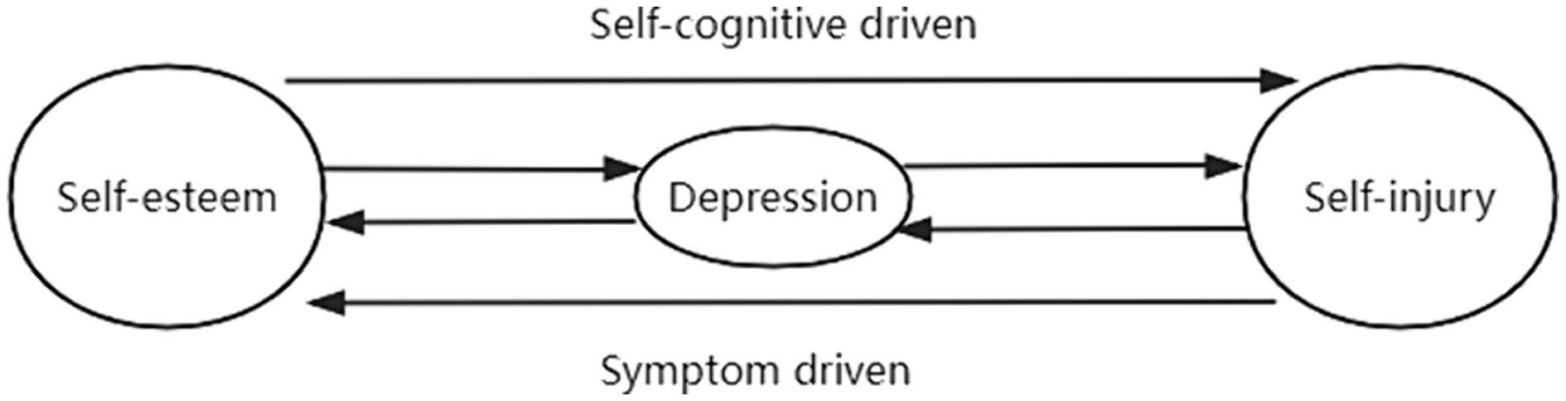
A model of depression-mediated cycles of self-esteem and self-injury.

In summary, based on the cognitive vulnerability model and the scar model, and assuming Hypotheses 2 and 3, this study proposes Hypothesis 4: There exists a dynamic cycle among an individual’s self-esteem, depression, and self-injury, characterized by two pathways: “self-esteem—depression—self-injury” and “self-injury—depression—self-esteem” (as shown in Figure 1).

## 2 Methods

### 2.1 Participants

Four junior middle schools in Zhangjiajie, Hunan Province, China, were selected to conduct three follow-up surveys by distributing questionnaires to students in the first and second grades at six-month intervals in the form of classes. The sample size of the first survey (T1) was 1,265, the sample size of the second survey (T2) was 1,207, and the sample size of the third survey (T3) was 1,173. Ninety-two subjects were lost due to withdrawal, suspension, and leave of absence, etc. After screening and deletion of 30 invalid samples, the sample of valid subjects adopted in this study was 1,143, and the effective follow-up rate was 90.36%. The baseline age of the subjects was 13.10 ± 0.93 years ranging from 11 to 15 years old, of which 538 (47.07%) were male and 605 (52.93%) were female. All participants and their guardians were required to provide written informed consent. This study underwent an ethical review and was approved by the Academic Committee of the College of Education of Hunan Agricultural University.

### 2.2 Measurements

#### 2.2.1 The childhood depression scale (CDI)

The scale was developed by Kovacs and revised by Wu et al. (19). It is applicable to ages 7–17 years old and consists of 27 items, including five dimensions: lack of pleasure, negative emotions, low self-esteem, feelings of ineffectiveness, and interpersonal problems. Each item has three options describing different degrees of depression, which are scored as 0–2, with higher scores indicating more severe depression. In this study, the Cronbach’s alpha coefficients for the three measurements were 0.889, 0.953, and 0.949, respectively.

#### 2.2.2 The self-esteem scale (SES)

Developed by Rosenberg in 1965 (20), this scale was originally used to assess adolescents’ overall feelings of self-worth and self-acceptance is the most widely used self-esteem measurement tool in China’s psychological community. The scale consists of 10 items, using a 4-point rating system where each item is scored from 1 (strongly disagree) to 4 (strongly agree). A higher total score indicates a higher level of self-esteem. In this study, the Cronbach’s alpha coefficients of three tests were 0.826, 0.893, and 0.902, respectively.

#### 2.2.3 The adolescent self-injury scale (ASIS)

The scale was compiled by Zheng and revised by Feng, with 18 entries to assess the frequency and severity of 18 common NSSI behaviors (21). The number of self-injuries was assessed as 0, 1, 2–4 and ≥ 5 times, with scores of 0–3, and the degree of injuries was categorized into five grades ranging from “none” to “extremely severe” with a score of 0–4, respectively. The overall severity of NSSI is equal to the sum of products of the frequency and severity of self-injuries. In the present study, the Cronbach’s alpha coefficients of three measurements were 0.937, 0.953 and 0.949, respectively.

### 2.3 Statistical analysis

Data were analyzed using SPSS 26.0 for common method bias test, descriptive statistics, correlation analysis, process 4.0 for cross-sectional mediation analysis, and Mplus 8.0 for cross-lagged statistical analysis. The K-S test for normality of the three times data of the three scales scores showed that the data did not conform to normal distribution, therefore, the robust maximum likelihood robust estimator (MLR) estimation method was used in constructing the cross-lagged model. In addition, previous studies as well as the present study showed significant gender differences in self-injurious behaviors among adolescents; therefore, gender was used as a control variable to analyze the data in the present study.

## 3 Results

### 3.1 Common method bias test

The Harman one-factor method was used to analyze the unrotated principal component factors of each topic simultaneously, and the results showed that the maximum factor variance explained by the 1st measurement extracted was 23.13%; the maximum factor variance explained by the 2nd measurement extracted was 26.69%; the maximum factor variance explained by the 3rd measurement extracted was 25.70%, which were less than 40%, which indicated that there was no serious common method bias in this study.

### 3.2 Descriptive statistics

An independent samples t-test of the total self-esteem, depression, and self-injury scores at the three time points showed that there were significant gender differences in self-esteem, depression, and self-injury at T1, T2, and T3, self-esteem scores were all significantly higher in boys than in girls, depression scores were all significantly higher in girls than in boys, and NSSI scores were significantly higher in girls than in boys (See Table 1).

**Table 1:** Gender Differences in self-esteem, depression, and self-injury test scale.

TIME	Genders	SES	CDI	NSSI
T1	Boy	29.690 ± 4.878	13.069 ± 7.643	4.881 ± 10.664
	Girl	28.330 ± 4.903	16.102 ± 8.673	6.867 ± 14.736
	t	4.689^***^	−6.284^***^	−2.631^**^
T2	Boy	29.432 ± 5.066	14.624 ± 8.137	5.931 ± 15.462
	Girl	27.682 ± 5.418	17.572 ± 9.014	7.932 ± 16.791
	t	5.618^***^	−5.810^***^	−2.097^*^
T3	Boy	30.185 ± 5.431	13.735 ± 8.633	4.050 ± 10.807
	Girl	28.394 ± 5.650	16.601 ± 9.070	5.986 ± 15.106
	t	5.448^***^	−5.454^***^	−2.512^*^

*p < 0.05, **p < 0.01, ***p < 0.001.

T1, time point 1; T2, time point 2; T3, time point 3; SES, self-esteem scale; CDI, children’s depression inventory; NSSI, non-suicidal self-injury.

### 3.3 Correlation analysis

Spearman rank correlation was used to correlate the data from the three measurement time points of self-esteem, depression and self-injury, and the results of the three correlation analyses are shown in Table 2. Self-esteem was significantly negatively correlated with depression at the three time points, and depression was significantly positively correlated with self-injury, and self-esteem was significantly negatively correlated with self-injury.

**Table 2:** Descriptive statistics and correlations of self-esteem, depression, and self-injury (*N* = 1,143).

	M	SD	T1SES	T2SES	T3SES	T1CDI	T2CDI	T3CDI	T1NSSI	T2NSSI	T3NSSI
T1SES	28.971	4.936	1								
T2SES	28.506	5.326	0.602**	1							
T3SES	29.237	5.618	0.475**	0.612**	1						
T1CDI	14.675	8.340	−0.673**	−0.585**	−0.498**	1					
T2CDI	16.185	8.734	−0.467**	−0.687**	−0.555**	0.644**	1				
T3CDI	15.252	8.978	−0.376**	−0.519**	−0.709**	0.529**	0.608**	1			
T1NSSI	5.933	13.012	−0.315**	−0.296**	−0.253**	0.434**	0.340**	0.278**	1		
T2NSSI	6.990	16.203	−0.223**	−0.348**	−0.300**	0.311**	0.427**	0.348**	0.528**	1	
T3NSSI	5.075	13.287	−0.180**	−0.291**	−0.342**	0.296**	0.341**	0.389**	0.444**	0.539**	1

**p < 0.01, ***p < 0.001.

T1, time point 1; T2, time point 2; T3, time point 3; SES, self-esteem scale; CDI, children’s depression inventory; NSSI, non-suicidal self-injury.

### 3.4 Cross-lagged analysis of self-esteem and self-injury

**Figure 2:**
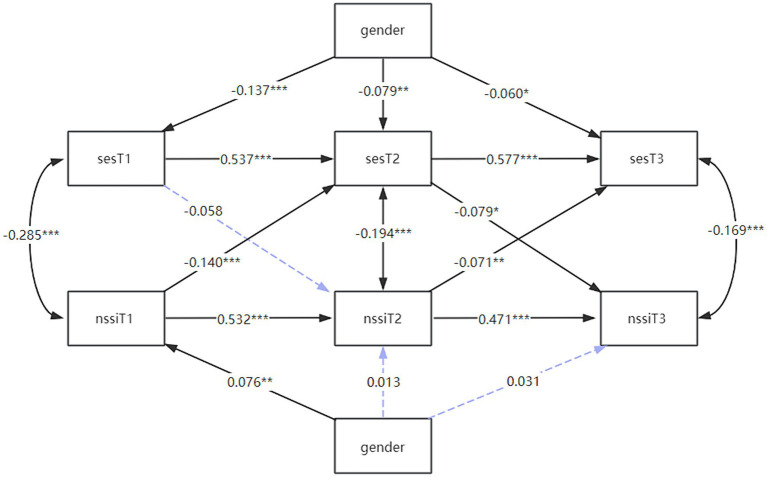
Cross-lagged model plot for self-esteem and depression. The figure shows the cross-lagged coefficients between self-esteem and depression at the three time points after gender was included as a control variable, with the blue dashed line representing no correlation. * *p* < 0.05; **p < 0.01, ***p < 0.001. T1, time point 1; T2, time point 2; T3, time point 3; SES, Self-esteem Scale; CDI, children’s depression inventory; NSSI, Non-suicidal self-injury.

To explore the interrelationship between self-esteem and self-injury, this study constructed a cross-lagged model of self-esteem and self-injurious behaviors with gender as a control variable, and the model fit indices were CFI = 0.956, SRMR = 0.030. The model results showed (Figure 2) that gender was a significant predictor of self-esteem at T1, T2, and T3; whereas gender was a significant predictor of self-injury only at T1, and not at T2 or T3. Self-esteem at T1 was a non-significant predictor of self-injurious behaviors at T2, whereas self-esteem at T2 significantly negatively predicted self-injurious behaviors at T3. Self-injurious behaviors at T1 significantly predicted self-injurious behaviors at T2, and the T2 self-injurious behavior similarly significantly negatively predicted T3 self-esteem. In other words, self-esteem and self-injury had a bidirectional predictive effect. These results partially supported hypotheses 1.

### 3.5 Analysis of the mediating role of depression between self-esteem and self-injury

Firstly, mediation effect analysis was conducted in cross-sectional survey with T1 self-esteem as the independent variable, T1 depression as the mediator variable, T1 self-injury as the dependent variable, and gender as the control variable. The results were shown in Supplementary Figure S1, self-esteem significantly negatively predicted depression, a = −0.669, *p* < 0.001; depression significantly positively predicted self-injury, b = 0.510, p < 0.001; self-esteem and depression entered the equation at the same time, and self-esteem did not significantly predict self-injurious behaviors c′ = 0.054, *p* = 0.1298. i.e., depression played the complete mediating effect in this model, and the value of the mediating effect was −0.669 × 0.510 ≈ −0.341 (BC95%CI [−0.401, −0.282]), *p* < 0.001.

**Figure 3:**
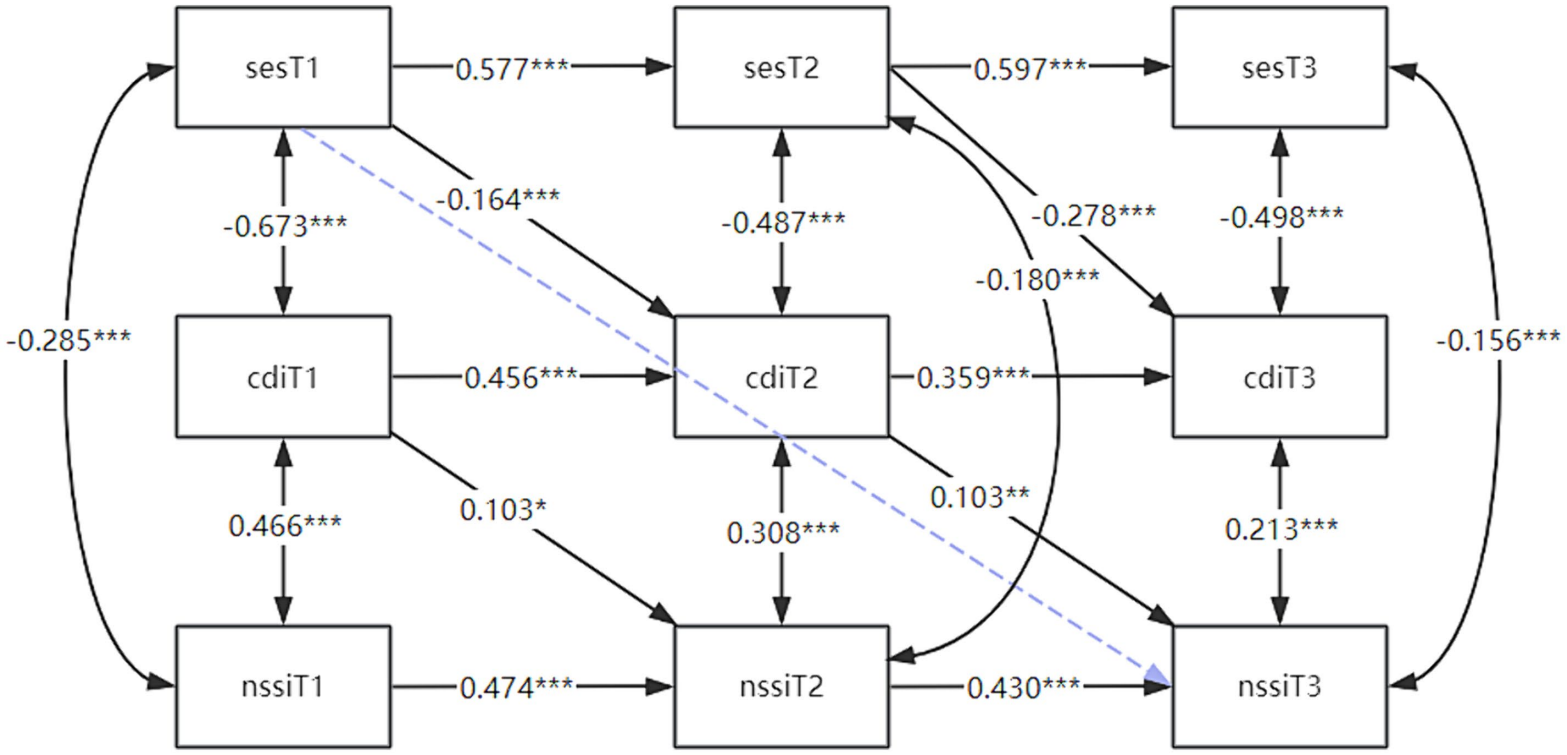
Longitudinal mediation model of depression between self-esteem and self-injury. The figure shows the crossover coefficients between self-esteem to self-injury at the three time points using depression as a mediator, with the blue dashed line representing no correlation. * *p* < 0.05; **p < 0.01, ***p < 0.001. T1, time point 1; T2, time point 2; T3, time point 3; SES, self-esteem Scale; CDI: children’s depression inventory; NSSI, non-suicidal self-injury.

On the basis of having verified that depression mediates horizontally between self-esteem and self-injury, a longitudinal mediation model was constructed to examine whether depression mediates longitudinally between self-esteem and self-injury, also using gender as a control variable. The respective fitting indices of the model were CFI = 0.925, SRMR = 0.074. As shown in Figure 3, self-esteem on T1 significantly negatively predicted depression on T2, and self-esteem on T2 significantly negatively predicted depression on T3; T1 depression significantly positively predicted self-injury on T2 and depression on T2 significantly positively predicted self-injurious behaviors on T3; and the direct predictive effect of self-esteem on self-injury on T3 was not significant for T1. In summary, there was a delayed predictive effect between self-esteem on depression and depression on self-injury, but the direct predictive effect of T1 self-esteem on T3 self-injurious behavior was not significant, which showed that T2 depression played a fully longitudinal mediating role between T1 self-esteem and T3 self-injury, with a mediating effect of −0.164 × 0.103 ≈ −0.017 [BC95%CI (−0.027, −0.007)], *p* < 0.01.

In summary, through both the cross-sectional and longitudinal analysis, the results indicated that the effect of self-esteem on self-injury was realized through the mediating variable of depression, i.e., depression mediated the relationship between self-esteem and self-injury, which supported hypothesis 2.

### 3.6 An analysis of the mediating role of depression between self-injury and self-esteem

To test hypotheses 3, mediation analyses were conducted with T1 self-injury as the independent variable, T1 self-esteem as the dependent variable, and T1 depression as the mediator, while gender was used as a control variable. The results, as shown in Supplementary Figure S2, showed that self-injury significantly predicted depression, *a* = 0.460, *p* < 0.001; depression significantly predicted self-esteem, *b* = −0.696, *p* < 0.001; self-injury and depression entered the equation at the same time, and self-injury was not a significant direct predictor of self-esteem *c*′ = 0.037, *p* > 0.05. i.e., depression played a fully mediating role in this model, and the mediating effect size was 0.460 × (−0.696) ≈ −0.320 [BC95%CI (−0.365, −0.275)], *p* < 0.001.

**Figure 4:**
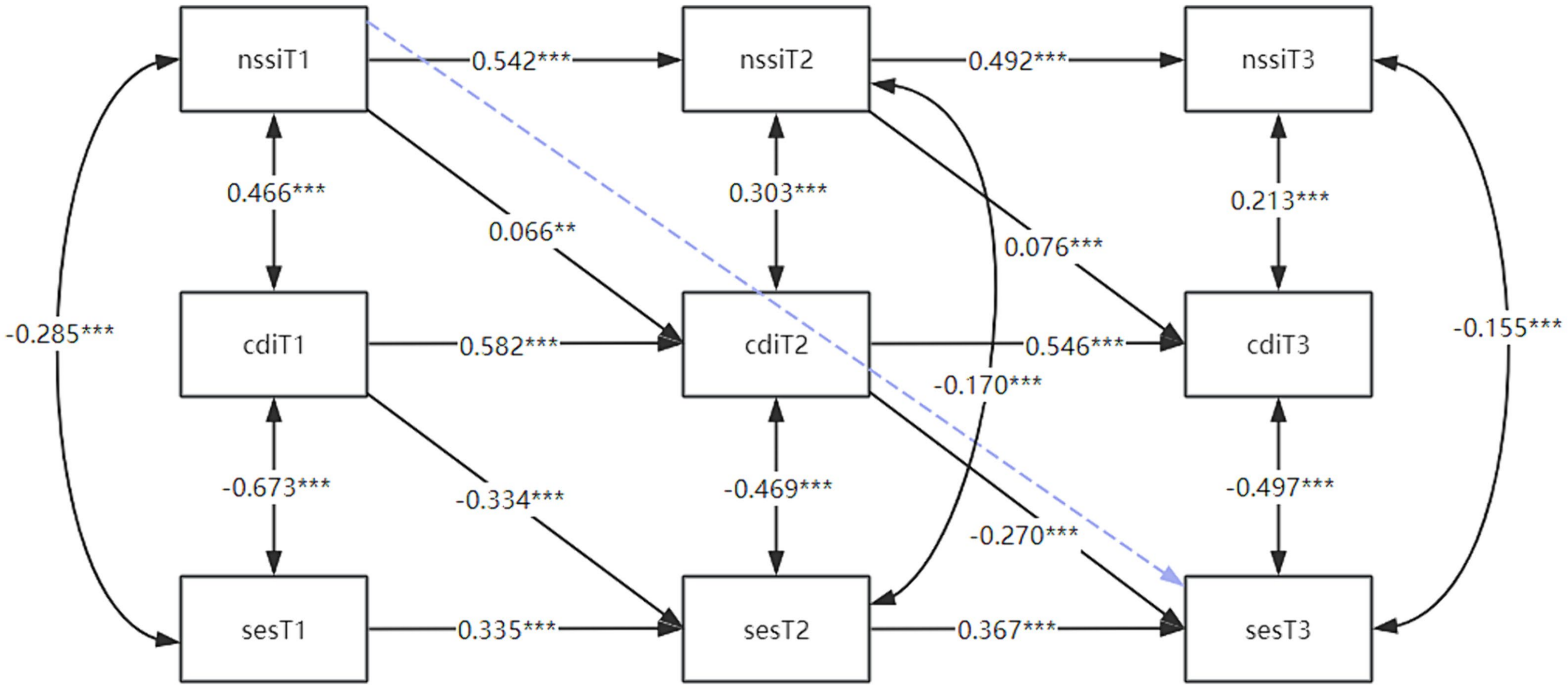
Longitudinal mediation model of depression between self-injury and self-esteem. The figure shows the cross-lag coefficients between self-injury to self-esteem at the three time points using depression as a mediator, with the blue dashed line representing no correlation. * *p* < 0.05; **p < 0.01, ***p < 0.001. T1, time point 1; T2, time point 2; T3, time point 3; SES, self-esteem scale; CDI, children’s depression inventory; NSSI, non-suicidal self-injury.

Similarly, this study examined whether depression mediates longitudinally between self-injury and self-esteem based on the fact that it has been verified that depression mediates horizontally between self-injury and self-esteem, and therefore constructed a longitudinal mediation model of depression between self-injury and self-esteem with a model fit index of CFI = 0.952 and SRMR = 0.064. The results of the model are shown in Figure 4. Self-injurious behavior (T1) significantly positively predicted depression (T2), and self-injurious behavior (T2) significantly positively predicted depression (T3). T1 depression significantly negatively predicted self-esteem on T2, and T2 depression significantly negatively predicted self-esteem on T3; and the direct predictive effect of T3 self-injury on T1 self-esteem was not significant. Thus, T2 depression fully mediated the relationship between T1’ self-injury and T3’ self-esteem with a mediating effect of 0.066 × (−0.270) ≈ −0.018 [BC95%CI (−0.029, −0.007)], *p* < 0.01. In summary, it can be seen that self-injury affects an individual’s subsequent depression as well as self-esteem, and that depression mediates the pathway self-injury-self-esteem. These results supported hypotheses 3.

### 3.7 An interactive cross-lagged model of self-esteem, depression, and self-injurious behavior

**Figure 5:**
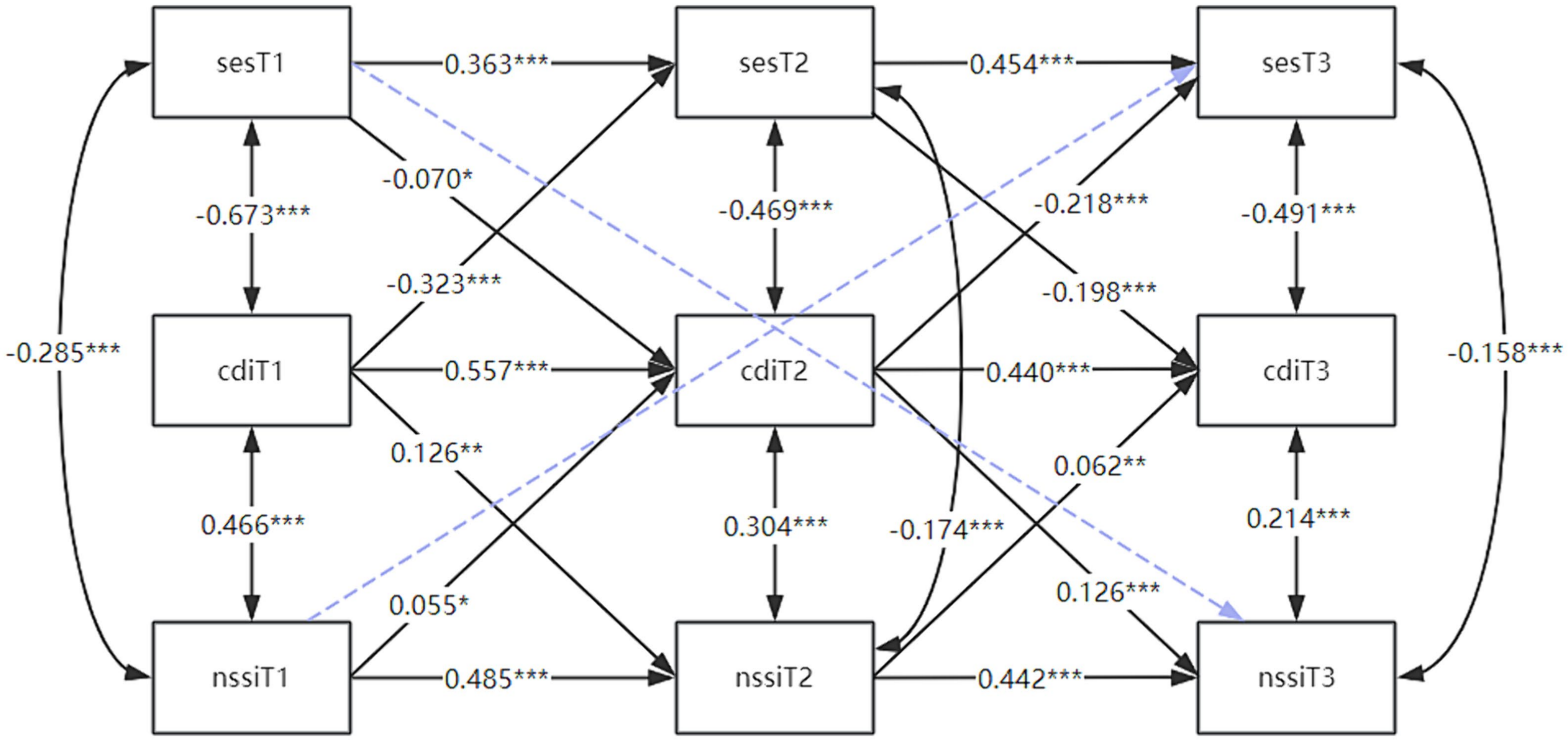
Cross-lagged modeling of the interaction of self-esteem, depression, and self-injury explores the reciprocal predictive relationships of self-esteem, depression, and self-injury. The figure shows the reciprocal cross-lag coefficients between self-esteem, depression, and self-injury at the three time points with depression as a mediator, and the blue dashed line representing no correlation. *p < 0.05; **p < 0.01, ***p < 0.001. T1, time point 1; T2, time point 2; T3, time point 3; SES, self-esteem scale; CDI, children’s depression inventory; NSSI, non-suicidal self-injury.

Lastly, this study also constructed a cross-lagged model of the interaction of self-esteem, depression, and self-injury behaviors to explore the reciprocal prediction of self-esteem, depression, and self-injury behaviors, with model fit indices of CFI = 0.962 and SRMR = 0.033, respectively. The results of the model are shown in Figure 5, which shows that self-esteem (T1) significantly negatively predicted depression (T2), and then depression (T2) significantly positively predicted self-injury (T3). self-injury (T1) was a significant predictor of depression (T2) which was a significant predictor of self-esteem (T3). Whereas the direct prediction of T1 self-esteem on T3 self-injury, and of T1 self-injury on T3 self-esteem were not significant. This showed that T2 depression plays a significant mediating role in both pathways: “T1 self-esteem toT3 self-injury” and T1 self-injury to T3 self-esteem.” In summary, there is a dynamic cycle between self-esteem, depression and self-injury, consistent with our H4 shown in Figure 1.

## 4 Discussion

This study used a longitudinal design to explore the relationship between self-injury, self-esteem, and depression in order to clarify the underlying mechanisms before and after the onset of self-injury in adolescents. According to the results of this study, girls scored higher than boys in both depression scores and self-injury scores, which is consistent with some previous studies (22–24). It proves that there is indeed a significant gender difference in the occurrence of self-injurious behavior and depression. Adolescence is in the critical period of individual physical and mental development, but due to the different rates of physical and mental development between boys and girls, girls are more susceptible to the influence of hormones and the living environment than boys, and are more likely to show high sensitivity and instability in self-perception and emotional fluctuations compared to boys. This may lead to girls being more likely to have emotional and behavioral problems.

In this study, the cross-lagged model was used to investigate the relationship between self-esteem and self-injury, and the results showed that self-esteem was able to predict self-injury within a certain period of time, and this result was the same as that of previous studies (6). Junior middle school students are in the critical period of academic development and physical and mental development, when they encounter unsolvable problems or receive unobjective evaluations, it will lead to negative perceptions of their self-competence, which will affect their self-esteem, and individuals with lower self-esteem are more likely to produce self-blame, and then adopt non-adaptive problem solving, that is self-injurious behaviors (25). Second, self-injury was a predictor of self-esteem, which is different from the results of previous studies (6). The results of this study suggest that persistent self-injurious behavior may lead to low levels of self-esteem, and self-injurers usually feel shame and self-blame for their behavior, which has a long-term negative impact on self-esteem, and the persistent self-injurious way of coping with the problem does not lead to a real solution, and it may even lead to the emergence of more mental health problems such as low self-esteem.

This study used a longitudinal design to explore the relationship between self-esteem, depression, and self-injury. The results of the study indicated that depression played a significant and fully mediating role between self-esteem and self-injury, both in the cross-sectional and longitudinal data. This finding is similar to that of Zhang et al. (26). In this study, it was found that shame and bullying play a mediating role between self-esteem and self-injury, suggesting that low self-esteem will lead to self-injury behavior by triggering shame and bullying in adolescents. Shame and depression are common negative emotions in adolescents, which are closely related to self-esteem. The onset of adolescence represents a peak in identity confusion and is a special period during which self-esteem levels tend to decline (27), while individuals with lower levels of self-esteem have more biased perceptions of the self and are unable to correctly recognize their self-worth, resulting in negative schemas. The cognitive vulnerability model of depression suggests that individuals with negative self-perceptions are more likely to adopt a pessimistic outlook on the future, leading to depression. The experiential avoidance model posits that after experiencing depressive emotions, individuals will seek ways to alleviate or escape these feelings. Given that adolescents’ cognitive development is still immature and influenced by their environment, their methods of relief may be limited, leading them to adopt non-adaptive coping mechanisms, such as self-injurious behavior. Therefore, low self-esteem in adolescents may lead to self-injury through the mediation of depressive emotions.

This study also examined the pathway from self-injury to depression to self-esteem, confirming that depression plays a full mediating role in this pathway. The study found that self-injury negatively predicts depression. This indicates that individuals’ self-injurious behavior does not truly liberate them from depressive emotions. Instead, self-injury may temporarily shift the individual’s focus from depressive emotions to physical pain, thereby temporarily alleviating negative emotions. According to the scar model, depression leaves a lasting scar, causing negative impacts on the individual and leading to a reduction in self-cognition, meaning that individuals experiencing depression are more likely to develop lower levels of self-esteem. It can be inferred that experiencing self-injury does not truly alleviate depression, and through depression, it impacts the individual’s level of self-esteem, leading to a reduction in self-esteem.

More importantly, this study validated the bidirectional predictive model of self-esteem, depression, and self-injury through interactive cross-lagged analysis. Firstly, low self-esteem can trigger high depression, which in turn can lead to self-injurious behavior. In this pathway, self-esteem, as a crucial self-cognition factor, leads to self-injurious behavior by triggering depressive emotions. Therefore, we name it the self-cognition driven pathway. Secondly, engaging in self-injurious behavior can increase depression levels, thereby reducing self-esteem. In this pathway, self-injury, as a non-adaptive behavior, increases depression levels, which in turn lowers individual self-esteem. Hence, we name it the symptom-driven pathway. Combining these two pathways illustrates the cyclical relationship between self-esteem and self-injury, with depression serving as a significant mediating factor. We name this model the depression-mediated self-esteem and self-injury cycle model, as shown in Figure 1.

This study has the following limitations. First, the subjects selected for this study were in early adolescence, and although previous studies have reported that this stage is a high prevalence of self-injurious behaviors, whether the results of this study can be generalized to other age stages such as late adolescence requires further research to confirm. Second, only three time points were measured in this study, thus only exploring the linear relationship between self-esteem, depression, and self-injury. In the future, more time points or ecological transient assessment methods can be adopted to explore the dynamic nonlinear relationship among the three variables. Third, the present study used a subjectively reported measure, which may be subject to recall bias and measurement error due to the individual’s emotional and psychological state at the time of measurement; therefore, a combination of self-assessment and other assessment (e.g., parents, teachers, peers, etc.) could be considered in the future. Forth, only the effects of self-esteem and depression on self-injury were considered in this study; other external (e.g., social support) or internal (e.g., mental toughness) protective factors could be considered in the future.

## 5 Conclusion

Adolescents’ self-esteem, depression, and self-injury are closely related; self-esteem and self-injury predict each other; self-esteem indirectly affects self-injury through depression; and self-injury indirectly affects self-esteem through depression. Based on the bi-directional prediction of self-esteem and self-injury mediated by depression, the present study proposed a theoretical model of the depression-mediated self-esteem and self-injury cycle. The model is useful for clarifying the psychological mechanisms of self-injury in adolescents, as well as for further understanding the effects of self-injurious behaviors on adolescents’ emotions and self-perceptions after the occurrence of self-injurious behaviors.

## Data availability statement

The original contributions presented in the study are included in the article, further inquiries can be directed to the corresponding author.

## Ethics statement

The studies involving humans were approved by the Academic Committee of the College of Education of Hunan Agricultural University. The studies were conducted in accordance with the local legislation and institutional requirements. Written informed consent for participation in this study was provided by the participants’ legal guardians/next of kin.

## Author contributions

HL: Data curation, Formal analysis, Investigation, Methodology, Writing – original draft. JX: Formal analysis, Methodology, Writing – original draft. YR: Investigation, Writing – review & editing. TZ: Formal analysis, Investigation, Writing – review & editing. XZ: Conceptualization, Funding acquisition, Investigation, Project administration, Resources, Supervision, Writing – review & editing.
