# Supplementary material for: Relationships among self-esteem, depression and self-injury in adolescents: a longitudinal study
Source: Front Public Health. 2024 May 15;12:1406283. doi: 10.3389/fpubh.2024.1406283 (PMC11135207; doi:10.3389/fpubh.2024.1406283)
Supplement: Supplementary file 1 [file Data_Sheet_1.docx]

**Supplementary Figure Legends**

**Figure S1.** Depression mediates the relationship between self-esteem and self-injury. The figure shows a cross-sectional mediation effect analysis done with T1 self-esteem as the independent variable, T1 depression as the mediator variable, T1 self-injury as the dependent variable, and gender as the control variable. In this model, depression played a full mediating effect.

Notes: * p<0.05; **p<0.01, ***p<0.001.

Abbreviations: T1=Time point 1; SES: Self-esteem Scale; CDI: Children's Depression Inventory; NSSI: Non-suicidal self-injury.

**Figure S2.** Depression mediates the relationship between self-injury and self-esteem. The figure shows a mediation effect analysis with T1 self-injury as the independent variable, T1 self-esteem as the dependent variable, and T1 depression as the mediator variable, as well as a cross-sectional mediation effect analysis with gender as the control variable. In this model, depression plays a fully mediating role.

Notes: * p<0.05; **p<0.01, ***p<0.001.

Abbreviations: T1=Time point 1; SES: Self-esteem Scale; CDI: Children's Depression Inventory; NSSI: Non-suicidal self-injury.

**Figure S1**

**
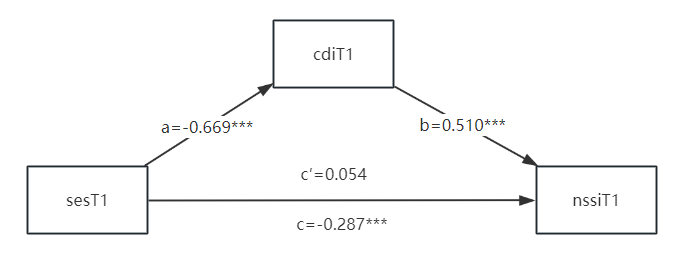
**

**Figure S2**

**
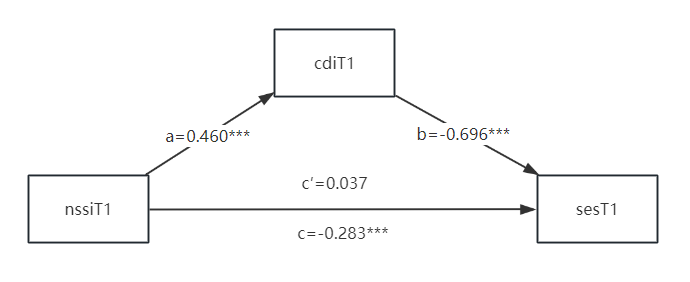
**
